# Long-Term In Vitro Assessment of Biodegradable Radiopaque Composites for Fiducial Marker Fabrication

**DOI:** 10.3390/ijms232214363

**Published:** 2022-11-18

**Authors:** Żaneta Górecka, Emilia Choińska, Marcin Heljak, Wojciech Święszkowski

**Affiliations:** 1 Division of Materials Design, Faculty of Materials Science and Engineering, Warsaw University of Technology, 141 Woloska Str., 02-507 Warsaw, Poland; 2 Centre for Advanced Materials and Technologies CEZAMAT, Warsaw University of Technology, 19 Poleczki Str., 02-882 Warsaw, Poland

**Keywords:** radiopacity, biodegradability, polyester, contrasting properties

## Abstract

Biodegradable polymer-based composite materials may be successfully utilised to fabricate fiducial markers (FMs), which are intended to precisely label tumour margins during image-guided surgery or radiotherapy. However, due to matrix degradability, the stability of the functional properties of FMs depends on the chosen polymer. Thus, this study aimed to investigate novel radiopaque composites which varied in the polymeric matrix—polycaprolactone (PCL), poly(L-lactide-co-caprolactone) (P[LAcoCL]) with two molar ratios (70:30 and 85:15), and poly(L-lactide-co-glycolide) (with molar ratio 82:18). The radiopaque component of the materials was a mixture of barium sulphate and hydroxyapatite. The changes in water contact angle, stiffness, and radiopacity occurring during the 24-week-long degradation experiment were examined for the first time. This study comprehensively analyses the microstructural causes of composites behaviour within degradation experiments using thermogravimetric analysis (TGA), differential scanning calorimetry (DSC), gel permitted chromatography (GPC), and scanning electron microscopy (SEM). The obtained results suggest that the utilized biodegradable matrix plays an essential role in radiopaque composite properties and stability thereof. This long-term in vitro assessment enabled a comparison of the materials and aided in choosing the most favourable composite for FMs’ fabrication.

## 1. Introduction

Fiducial markers (FMs) are radiopaque implants, a few millimetres in size, that are utilized for soft tissue tumour marking during image-guided radiotherapy and in follow-up diagnostics [1,2]. It is assumed that FMs should fulfil their function by six months, after which their presence in the cured site may negatively interfere with future diagnostics. Thus, the currently used nondegradable metallic FMs should be removed as, additionally, they may cause discomfort or pain due to too high a stiffness [3,4,5]. However, if the material of the FMs was biodegradable and possessed a lower stiffness, the adverse effects of metallic FMs could be eliminated. Moreover, FMs require appropriate anchorage in the tissue to perform their function of being reference points. This can be obtained by using a specific macroscopic shape. However, surface properties are also crucial for cell adhesion. It is known that cell attachment is promoted on more hydrophilic surfaces [6,7].

The alternative for metallic FMs might be biodegradable polymer-based composite FMs. However, since the X-ray attenuation of polymers is in the range of soft tissue, the composite should contain a radiopaque filler to generate contrast in soft tissue surroundings. Moreover, the composite concept enables the tailoring of contrasting properties because the content or the type of fillers may be adapted [3].

The fillers that have already been tested in the literature are FDA- and EMA-approved inorganic salts, such as barium sulphate (BaSO_4_) [3,8,9,10], as well as highly water-soluble iodinated organic compounds, e.g., iohexol or iodixanol [10,11]. Moreover, there is also an increasing interest in the utilisation of more biocompatible fillers, offering a good contrast in soft tissue, such as calcium phosphate ceramics, e.g., hydroxyapatite (HAp) [6,12,13] or HAp modifications [14,15,16]. Even though various fillers were singly investigated, the novel composites containing notable filler content are currently in development and should be comprehensively evaluated.

Many biodegradable polyester-based composites have already been tested in numerous applications for implantable devices [10,17,18]. Frequently utilised matrixes were homopolymers and copolymers of lactide, caprolactone, or glycolide. Depending on the composition, these polymers differ, e.g., in degradation time and in mechanical and thermal properties, which affect the functional properties and stability thereof in body conditions [19,20]. Some biodegradable polymers were also tested for radiopaque composites for implants [9,18,21,22]; however, the published reports mainly focused on the prepared composites’ initial properties. There is still a lack of long-term observations regarding the functional properties of many contrasting composite materials.

Thus, this study aimed to investigate the long-time effect of the biodegradable polyester matrix composition on the functional properties of novel radiopaque composites. The first, polycaprolactone (PCL), known to have a slow degradation rate (2–3 years), is used as a stable reference matrix [20]. The next two, copolymers of L-lactide, with caprolactone having molar ratios of 70:30 and 85:15, are favourable for soft tissue applications [23,24] and possess more rapid degradation rates and lower stiffness than the poly(L-lactide) or poly(caprolactone) [20,23]. Moreover, the copolymer of L-lactide with glycolide, with a molar ratio of 82:18 and a degradation rate of approx. 5–6 months, was investigated [20]. The sub-micron BaSO_4_ and nanoparticulate HAp mixture was chosen as a stable contrast filler system [6,13]. Composites were prepared via the solvent casting method. Subsequently, the prepared materials were thermally processed via a micro-extrusion process. The extruded model FMs in the form of rods with a 1 mm diameter were used in a 24-week-long degradation experiment performed in phosphate-buffered saline (PBS) at a pH of 7.4, which simulated the physiological conditions. The stability of the contrasting properties of the composites was controlled via Micro-Computed Tomography (MicroCT). The elastic modulus change was evaluated at 37 °C in wet conditions. Water contact angle (WCA) measurements and scanning electron microscopy (SEM) were performed to assess the surface microstructural changes caused by the degradation of materials. Parameters such as mass loss of samples, water absorption, and the pH change of PBS were evaluated at each time point to better understand the materials’ behaviour. Moreover, the content of fillers before and after degradation was assessed by thermogravimetric analysis (TGA). The molecular weight (MW) distribution, characteristic temperatures, and crystallinity of the prepared composites were evaluated using gel permitted chromatography (GPC) and differential scanning calorimetry (DSC), respectively. The performed analysis enabled a comparison between radiopaque biodegradable composites for the first time and a correlation of the matrix characteristics with the functional properties of the resulting composites.

## 2. Results

### 2.1. Fabrication of Experimental Samples

**Figure 1:**
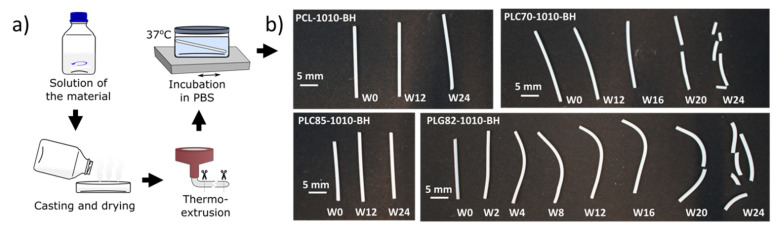
(**a**) Procedure of samples’ preparation; (**b**) photography of the samples after incubation in PBS at 37 °C.

The four commercially available polyesters (PCL, PCL70, PLC85, and PLG82) used in this study to prepare composite materials were already widely tested in the literature for various implantable applications [25,26,27,28]. However, after combining these polymers with a mixture of BaSO_4_ and HAp in the proportion of 8:1:1 (polymer:BaSO_4_:HAP), new materials were created that provided radiopacity in the range of bone (Figure S1). The fabrication process of the model FMs, using 1 mm-diameter rods, was performed in two steps: preparation of the composite using a solvent casting technique and the thermal processing of the materials (Figure 1a). 

The results of the TGA, GPC, and DSC at each step of the rods’ fabrication were presented in Table 1. Briefly, the desired amount of the inorganic phase (20 wt%) was obtained for each composite material, which was revealed by TGA. The performed GPC analysis showed that the preparation method of composites caused only minor changes in the MW distribution of tested polymers. Nevertheless, from the results of the second heating cycle of DSC (Table 1 and Figure S2 in Supplementary Materials), it can be observed that the addition of powders accelerated the crystallisation kinetics of the matrix. Polymeric chains could crystalise at lower temperatures, resulting from the nucleation of crystallites on the fillers’ particles [29,30]. Moreover, the enthalpy of the crystalline phase in composite matrixes was higher than in the raw polymer. These findings are consistent with the previous reports on BaSO_4_ and HAp in polyester matrixes [29,30,31].

In the additional step, the prepared composites were thermally processed by micro-extrusion using BioScaffolder. Although there was the possibility to extrude the obtained materials, e.g., the PCL-based composite through the 0.10 mm nozzle (Figure S3), the 1 mm-diameter rods extruded via a G17 nozzle were prepared as a reference to commercially available FMs [32,33,34].

The thermal processing caused changes in the MW distribution of all tested materials, which was also reflected in the changes in the thermal characteristics. The determined values of particular GPC and DSC parameters of thermally extruded rods can be found in Table 1. The observed changes in DSC data resulted from the higher mobility of the shorter macromolecules, which, in the rubbery state of polymer-facilitated crystallisation, led to increased crystallite size and/or perfection and elevated the crystalline phase content [35]. The plausible main phenomena causing the polyester chains’ cleavage during thermal processing are: (1) the thermal degradation connected with the spontaneous random chains scissions and/or *-co-* bond breakage [36], (2) the thermo-mechanical degradation connected with the stretching of chains due to the shear stress [37,38], and (3) transesterification, e.g., between the -OH group on the HAp surface and the -COO- group of the polyester chain [29]. Nevertheless, after comparing the tested materials, a more pronounced effect of thermal processing was observed for the copolymeric matrixes than for polycaprolactone.

### 2.2. Degradation Experiment

All composites in the form of rods were subjected to 24-week-long incubation in PBS at 37 °C (Figure 1a) to assess the changes occurring in the materials in the physiological conditions. A visual observation of the rods (Figure 1b) did not reveal the changes in the PCL- and PLC85-based composites. However, for the PLC70- and PLG82-based composites, the fragility of the dried samples was observed at the end of the experiment. Moreover, for PLG82-1010-BH, the deformation of rods due to the angular samples’ placement in the incubation container (well of 12-well plate) was observed from W2.

### 2.3. Mass Loss

**Figure 2:**
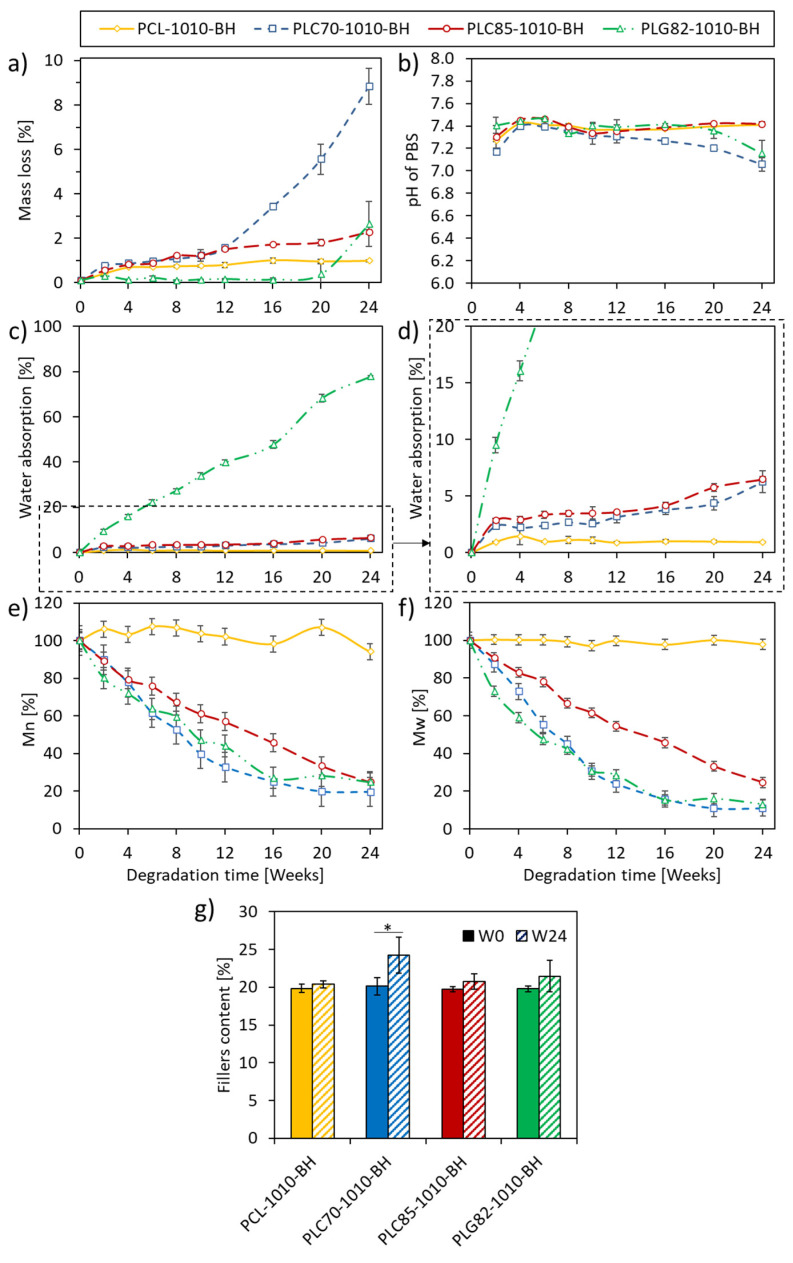
Results from degradation experiment of composites: (**a**) the mass loss, (**b**) pH of PBS, (**c**,**d**) the water absorption, (**e**) the relative $M_{n}$, (**f**) the relative $M_{w}$, (**g**) the content of fillers in the matrix measured by TGA. *—when *p* < 0.05.

The mass loss of all investigated types of rods was presented in Figure 2a. At the first time interval, the cumulative mass loss was related to the monomers’ and oligomers’ fraction release [19,39]. For the PCL-based composite, the mass loss at W2 reached 0.3%, whereas, at W24, it was 0.9%. For the PLC85-based composite, a more robust trend was observed: the mass loss at W2 and W24 reached 0.5% and 2.2%, respectively. Within the first 12 weeks of the experiment, a negligible difference was observed between PLC70-1010-BH and PLC85-1010-BH (both obtained approx. 1.5% at W12). However, a sudden increase in mass loss occurred for PLC70-1010-BH after W12. Finally, it reached 9% at W24. In the case of the PLG82-based composite, an almost constant mass was observed until W20; at the last time point (W24), a substantial mass loss of up to 2.5% was recorded.

### 2.4. pH of PBS

The initial pH decrease (at W2, Figure 2b) of PBS occurred for all investigated composites and was related to the release of monomers and oligomers [19,39]. However, within the next ten weeks, the pH of PBS remained almost constant (pH 7.4). For PCL- and PLC85-based composites, the pH value remained at the same level until the end of the experiment; for PLG82-1010-BH, it was until W16. For the PLC70-based composite, the pH decreased from W16, which was correlated to the mass loss. A similar observation was made for PLG82-1010-BH from W20. Nevertheless, the recorded values did not exceed pH 7.

### 2.5. Water Absorption

The investigated materials represented three levels of water absorption (Figure 2c,d). For the PCL-based composite, water absorption was lower than 1% during the experiment; PLG82-1010-BH steadily reached almost 80% at the end of the experiment. Both P[LAcoCL]-based composites had similar water absorption levels (approximately 6.5% at W24); however, it was slightly higher for PLC85-1010-BH than for PLC70-1010-BH.

As was observed, composites containing a copolymeric matrix facilitated higher water absorption. The reason for this was the higher ester group content per length unit in copolymers containing lactide and the glycolide repeating unit than in polycaprolactone, making them more hydrophilic [40].

### 2.6. MW Loss

There was no more than 5% of MW change during 24 weeks of the experiment for the PCL-based composite (a sudden increase in $M_{n}$ by 5% at W2, then a gradual decrease to approx. 95% of initial state). Whereas, for composites with copolymeric matrixes, the total MW loss was more than 75% (Figure 2e,f). The change of PLC85-1010-BH decreased linearly to approx. 25% of both initial averages of MW. For the PLC70- and PLG82-based composites, the decrease in MW had a similar and more exponential-like character. At W24, the average MW values for PLC70 and PLG82 matrixes reached approx. 19% and 24% of the initial $M_{n}$ and 11% and 13% of the initial $M_{w}$, respectively. 

### 2.7. Filler Content 

Within the degradation experiment, the content of the filler, calculated as a residue at 600 °C in TGA analysis, increased significantly for PLC70-1010-BH. The average value also increased for other materials, albeit without a significant difference (Figure 2g). The increased filler content, which corresponded with the material mass loss, indicated the matrix’s degradation and release of degradation products. 

### 2.8. Thermal Properties

**Figure 3:**
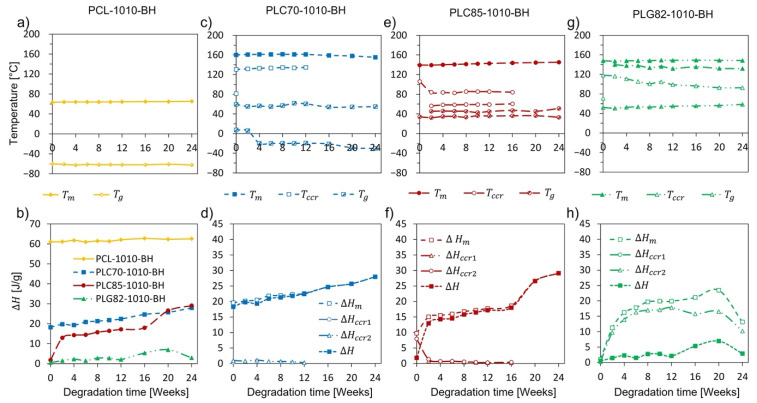
The characteristic temperatures (**a**,**c**,**e**,**g**) and enthalpies (**b**,**d**,**f**,**h**) determined from first heating of DSC for: (**a**,**b**) PCL-1010-BH, (**c**,**d**) PLC70-1010-BH, (**e**,**f**) PLC85-1010-BH, (**g**,**h**) PLG82-1010-BH.

During the degradation experiment, no explicit changes were observed in the thermal characteristics of the PCL-based composite. The characteristic temperatures and enthalpy recorded in both heating cycles remained almost constant (Figure 3a,b, Figures S4a,b and S5a,b).

For the copolymer-based composites, more remarkable changes occurred. The results of the DSC of copolymer-based rods are presented in Figure 3c–h, Figures S4c–h and S5c–h. The matrixes of the PLC70-1010-BH and PLC85-1010-BH composites were in a rubbery state at the human body temperature, whereas PLG82, the matrix of PLG82-1010-BH, was in a glassy state. The observed $T_{g}$ decline for PLC70-1010-BH during the degradation experiment reflected an increase in the polymeric chains’ mobility caused by scission thereof [35]. In the case of PLG82-1010-BH, the $T_{g}$ progressively increased with incubation time. Moreover, for this material, at the glass transition temperature range, a strong relaxation of the signal containing two little overlapping endothermic peaks occurred (Figure S4g), plausibly originating from the melting of the arranged low-MW, fraction-like monomers and oligomers [41]. The overlapping endothermic peaks became more prominent during the degradation experiment, and the maxima thereof shifted to higher temperatures. For all composites, crystalline content increased, resulting from microstructural changes caused by the reorganisation of macromolecules after preferential degradation in the amorphous region [42]. A detailed description of the results can be found in Supplementary Materials—Section S2. Detailed DSC analysis. 

### 2.9. Contrasting Properties

**Figure 4:**
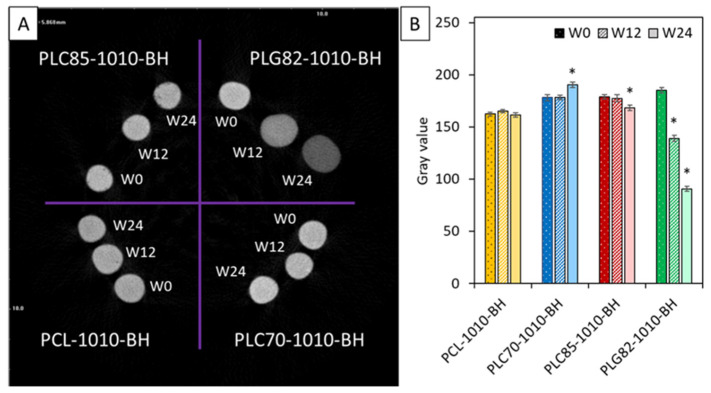
MicroCT results: (**A**) reconstructed cross-section image of tested samples, (**B**) average values of signal intensity in greyscale versus types of materials during degradation. *—when *p* < 0.05 compared to the initial state (W0).

In Figure 4, the MicroCT results of the investigated composites were presented. The qualitative analysis of radiopacity was performed by calculating the average grey value. The radiopacity of PCL-based composite rods was constant within the experiment period. For PLC70-1010-BH, it increased at the end of the experiment. Contrastingly, for PLC85-based rods, it slightly decreased at W24. For PLG82-1010-BH, a steady decrease was observed at each investigated time point.

### 2.10. Mechanical Properties

**Figure 5:**
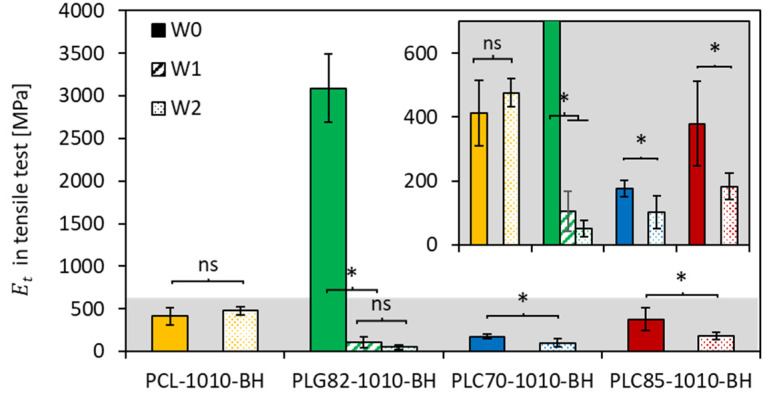
Results of $E_{t}$ from tensile test performed in PBS at 37 °C (insert presents a range of 0–700 MPa); *—when *p* < 0.05, ns-when *p* ≥ 0.05 in post hoc one-way ANOVA with a Tukey–Kramer pair-wise comparison test.

The stiffness of the materials was investigated in wet conditions (PBS) at 37 °C to assess the behaviour of the simulated physiological conditions. The obtained results (Figure 5) showed that, for PCL-based composite rods, the average $E_{t}$ value only insignificantly increased within two weeks. In the case of all copolymer-based composites, after a certain time of incubation, the obtained $E_{t}$ differed significantly from the initial state. For PLG82-1010-BH, which evidently possessed the highest initial stiffness (3089 ± 397 MPa), the $E_{t}$ at W1 decreased by approx. 30-fold (105.5 ± 62.3 MPa); at W2, it was even smaller (50.7 ± 26.7 MPa). In the case of the PLC70-based composite, within two weeks of incubation, the $E_{t}$ decreased from 175.5 ± 25.3 MPa to 101.9 ± 51.6 MPa; for PLC85-1010-BH, it was from 379.4 ± 132.4 MPa to 183.4 ± 40.4 MPa.

### 2.11. Surface Properties

**Figure 6:**
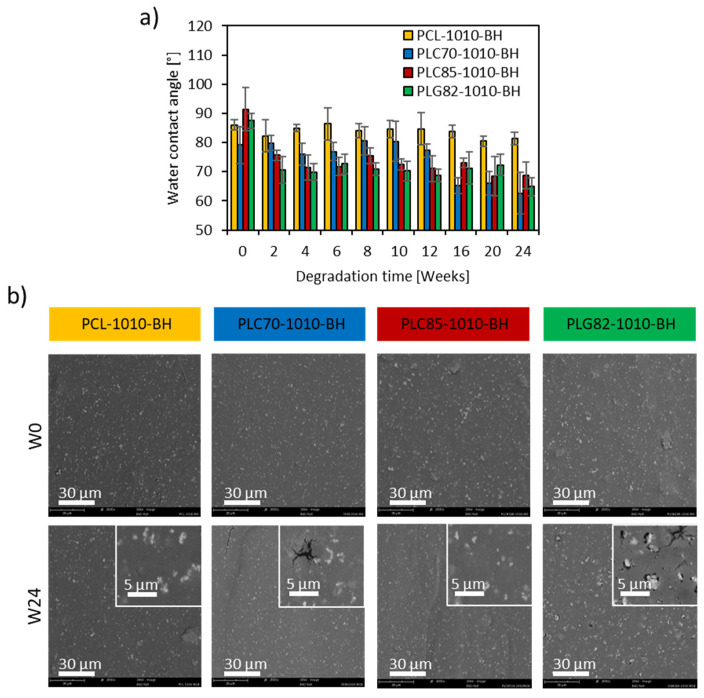
(**a**) WCA within the degradation experiment; (**b**) morphology of the surface of composite rods at W0 and W24.

The change in the surface wettability of the composites, which plays a crucial role in the cell-biomaterial interactions [6,7], was assessed by static WCA measurements. At the initial state, no significant difference between PLG82- (87.5° ± 2.6°), PCL- (86.0 ± 1.8°), and the PLC85-based (91.3 ± 7.4°) composites was observed (Figure 6a). The value of PLC70-1010-BH (79.2° ± 6.3°) was significantly lower than for other groups. Moreover, it remained at a similar level until W12; at W16, it significantly decreased to approx. 65° and remained constant until the end of the experiment. The WCA for other copolymer-based composites at W2 swiftly decreased to approx. 70–72° and remained almost constant until the end of the experiment. The WCA did not change remarkably for PCL-based composite during the whole experiment. 

The SEM images showed no difference between the topography of the samples at W0 (Figure 6b). Moreover, the morphology of the surface of the PCL- and PLC85-based composites did not change significantly during the degradation experiment. Some erosion symptoms on the surface of the samples were found only in the case of the PLC70- and PLG82-based composites. The cracks and infrequent pores in the matrix appeared in PLC70-1010-BH from W20. However, for PLG82-1010-BH, the plentiful pores at the interface of fillers and the matrix become gradually evident from W8. Moreover, after the infrequent loss of filler particles, the voids were observed at the last time point.

## 3. Discussion

A six-month period, which covers resection, radiotherapy, and further diagnostics during adjuvant therapy, is recommended for precise tumour margin labelling with implanted FMs [1,2,4,5,43]. However, after fulfilling the function, there is no need to maintain the FMs in the cured site as they could cause interference during future diagnostic imaging [3,4] or lead to discomfort or pain if their stiffness is too high [1,3]. Therefore, utilising biodegradable material for fiducial marker fabrication is crucial for avoiding excessive surgical interventions [3,6,13].

Biodegradable polymer-based composites utilised for FMs’ manufacturing also possess additional advantages. Firstly, the composition of such materials can be easily adapted for good visualisation in various medical imaging techniques [44]. In X-ray-based imaging, the physiological range of the attenuation (which corresponds with the air-bone attenuation window) is the most valuable for clinical analysis [3]. Moreover, the stiffness of polymeric materials is in the range of body tissues, decreasing mechanical irritation [24,45]. Another advantage is that polymeric composites can be easily formed in typical industrial polymer processing and postprocessing technologies [46]. Thus, a specific macroscopic shape combined with proper surface properties that enable soft tissue attachment or ingrowth can be easily achieved, providing a crucial feature of FMs—anchorage in the tissue leading to stability of reference points within the treatment time [6].

This study investigated four thermoplastic biodegradable polyesters proposed for the matrixes of radiopaque composite materials dedicated to FMs’ fabrication. A similar composite based on the PLC70 matrix containing BaSO_4_ and HAp was already reported regarding cytotoxicity and cell adhesion [6,13]. In this study, the characteristics of the tested materials were performed regarding long-term in vitro incubation in physiological conditions.

Initially, the molecular weights of the tested polymers were similar. The composites preparation method did not change the MW distribution appreciably; however, it allowed for the interactions between the polymeric chains of all types of the polymeric matrixes and the fillers’ surface, which was revealed through Fourier transform infrared (FTIR) analysis (see supplementary data—Section S3. FTIR analysis of filler–polymer interactions). Moreover, due to these interactions, the fillers affected the crystallisation kinetics of the matrixes [30,47]. The increased ΔH/w revealed a higher crystalline phase content, which correlates with materials stiffness and decelerates the material’s degradation [40,48].

The interactions also led to the formation of a polymeric layer on particles at the surface of an extrudate, which was evidenced by XPS in our previous study [6]. Thus, during the incubation experiment, the fillers washing away from the bulk or surface of the materials was inhibited (shown by SEM analysis). Additionally, as a result of the low solubility of both fillers in water [6,49,50,51], the physical mass of the filler remained constant over time while the reduction in the polymer matrix occurred, causing an increase in the content of the analysed fillers. Nevertheless, the stability of the fillers in the matrixes enabled the assessment of the effect of the matrixes’ microstructural changes occurring during incubation in the physiological conditions of the functional properties of the composites [17,25]. However, in case of the in vivo implantation of the tested materials, after matrix degradation, the released fillers should be phagocytosed and removed from the implantation site [52,53,54].

### 3.1. PCL-Based Composite

The PCL-based composite was stable during the experimental condition. The reason for the slow surface degradation of PCL and its hydrophobic character [40] was the high WCA and low water absorption rate, obtained and evidenced during the degradation experiment. No noteworthy surface morphology or wettability changes were observed, indicating slow degradation. The increase in E reported in the mechanical test results was the result of slightly increased crystalline phase content and the growth or higher perfection of the crystallites noted in the first heating of DSC through changes in $H_{m}$ and $T_{m}$, respectively [30,31]. The contrasting properties of this composite also did not change as the material composition (filler content) was constant. Based on the obtained results and previous reports, it can be concluded that the stability of the PCL matrix could be maintained for much longer than six months [20,55]; thus, the functional properties could be provided for longer than necessary for FMs.

### 3.2. PLC70-Based Composite

Within the first half of the experiment, the PLC70-1010-BH rods’ degradation occurred macroscopically imperceptible. The decreased pH of PBS at W2 occurred due to the elution of residual monomers created during thermal processing. Even though the majority of the measured parameters remained stable within the first half of the experiment, the average MW quickly decreased (to 33% of initial $M_{n}$ and 24% of initial $M_{w}$ at W12), indicating the bulk mechanism of degradation and leading to the rearrangement of polymeric chains. However, this did not affect the contrasting properties or the surface wettability within 12 weeks of the experiment. At the second half of the experiment, the observed changes in these properties were linked with advanced material degradation. As a result, a drop in WCA was observed from W16. Moreover, from W20, the fragility of rods, cracks, and infrequent pores on the surface of the PLC70 matrix occurred. The reduction in the MW, combined with the almost undetectable $T_{ccr}$ (in first heating cycle of DSC), proved the matrix crystallisation easiness that led to the gradual crystallinity degree enhancement. Nevertheless, the synergistic effect of elevated temperature, the presence of water molecules in the material, and the accumulation of the low-MW chains of polymer, acting as plasticizers, caused the reduction in material stiffness that was measured after two weeks of incubation. This phenomenon could be advantageous in vivo as materials that are too stiff irritate the surrounding tissues [24,45].

Additionally, the progressive degradation of the matrix affected the contrasting properties of the PLC70-based composite. The MicroCT reconstructions showed a slight increase in radiopacity at W24. This could be linked with the enhanced density caused by increased crystalline phase content and/or matrix degradation and the fillers’ increase in content. However, if other contrast agents with tailorable release from the matrix would be utilised, the stability of the radiopacity could be adapted [13]. The obtained results indicate progressive material degradation; however, the rate of the changes provides the stability of the contrasting properties within the needed six months. Further bulk decomposition of the matrix should lead to contrasting agents’ release and progressive contrast vanishing, which is required for the analysed application. 

### 3.3. PLC85-Based Composite

Compared to PLC70-1010-BH, the PLC85-based composite demonstrated almost the same mass loss and water absorption within 12 and 24 weeks, respectively. Moreover, for PLC85-1010-BH, the initial pH drop due to the residual monomers elution was also recorded [19,39]. However, further incubation time did not affect the mass loss rate or pH change, even though the MW reduction occurred progressively, indicating a bulk degradation mechanism. The most noticeable changes in the PLC85-based material microstructure occurred within the first time interval (at W2). A remarkable crystallinity increase at the incubation conditions was caused by the synergistic effect of the short chains’ presence and the incubation temperature—approaching the $T_{g}$, which facilitated the rearrangement of the macromolecules [35]. However, the comparison of the noted E values at W0 and W2 in wet conditions revealed a decrease in the material stiffness, which was still remarkably higher than those for the PLC70-based composite. Further incubation time led to a slight increase in crystalline fraction content up to W16, after which the second elevation of crystallinity degree was noted. The average MW loss exceeded 50% at this point, facilitating the further macromolecules’ arrangement. As a result, in the PLC85 matrix, the crystallinity fraction at W24 was lower than for PLC70 (approx. 45% and 53%, respectively), which was related to a larger, hardly crystallisable lactide fraction in the copolymer [40,56,57].

Within the degradation experiment of PLC85-1010-BH, no remarkable change in the surface morphology was noted by SEM analysis; however, the wettability decreased at the first time interval and remained at the same level until the end of the experiment. The radiopacity of PLC85-1010-BH was relatively stable within 24 weeks of the experiment and should provide the needed contrasting properties.

### 3.4. PLG82-Based Composite 

In the case of the PLG82-1010-BH, the progressing bulk degradation was macroscopically observed. The evidence for this was the intense swelling, deformation, brittleness, and loss of integrity of the samples at the end of the experiment. The performed examinations revealed extensive water absorption, which exceeded more than 16-fold the values obtained for other investigated composites at the end of the experiment. The swelling of the matrix caused the appearance of voids at the filler–matrix interface, which decreased particles’ anchorage. This led to the partial loss of particles from the surface and thus to the erosion of the material. Concurrently, the MW loss and bulk degradation were observed, which led to the appearance of infrequent pores in the matrix.

Furthermore, despite a substantial drop in WCA at W2 and the microscopically observed voids at the filler–matrix interface from W8, the mass of the PLG82-1010-BH samples and the pH of PBS were constant until W20. This could be caused by intermolecular forces which trap hydrolysis products inside the material [58]. As a result, a sudden rise in mass loss and decrease in pH occurred only at the final time point. Moreover, as the content of the introduced fillers remained constant, it can be assumed that the material composition did not change within degradation. 

Additionally, as could be observed in the DSC analysis, the PLG82-based rods were practically amorphous (ΔH/w < 9 J/g at W20, which gives approx. 10% of crystalline degree originating from lactide fraction) during the entire experiment. However, in first run of the DSC, the additional endothermic peaks were observed in a range of 60–80 °C, just above the glass transition. These peaks originated from the melting of the arranged low-MW fraction, such as oligomers [41], which supports our finding that they were observed only after thermal processing (causing an increase in PDI) and became more pronounced within the degradation experiment. The appearance of low-MW fraction and its amplification in the material was evidenced by a negligible mass loss of PLG82-based rods and a lack of decline in the pH of PBS until W20, whereas the MW decreased remarkably. The low release of low-MW fraction had to be caused by an arrangement thereof inside the matrix. This statement is supported by the growth of the arranged structures within the degradation, which was reflected by the additional endothermic peak shift to higher temperatures and the enhancement of its area. Our finding is concise with previous reports suggesting the existence of weak intra- or intermolecular interactions, including hydrogen bonding, in polyesters such as PCL, PGA, and P[LAcoGA] [48,59,60,61]. These interactions trigger higher-order structures, such as lamellar structures, and significantly impact the physical properties of polymeric materials [62,63].

The accumulation of the degradation products inside the PLG82 matrix led to the stability of pH value until W20 [59]. However, at W24, an immediate pH drop occurred, which complied with mass loss and suggested the starting point of enormous material erosion and remarkable acidic product release. The consequential unfavourable effect on the surrounding tissues could lead to a robust inflammatory response in vivo [63].

Considering the PLG82 as a matrix for composite FMs, it must be noted that the filler packing density decreased due to the matrix’s swelling. This worsened the contrasting properties. Moreover, the swelling also had an impact on mechanical properties. In wet conditions, the water uptake combined with the plasticizing effect of absorbed water molecules caused a more than 30-fold decrease in stiffness. Even though the mechanical properties of the PLG82 matrix may be favourable for soft tissue applications, the stability of the contrasting properties, immediate pH drop, and release of degradation products after six months is not satisfactory. 

Based on our findings, it can be concluded that the functional properties of composites with polymeric matrixes characterised by high stability in simulated physiological conditions, such as PCL or PLC85, may be maintained for much longer than six months. This feature could be favourable for long-term contrasting devices. However, in the case of FMs, it is crucial to avoid interference during further diagnostic imaging [5,6] or surrounding tissue irritation [3,5], as such prolonged contrasting properties are undesired. Adversely, the stability of contrasting properties for the PLG82 matrix, which reveals swelling behaviour, was not assured within the necessary time. Moreover, the delayed release of the degradation products of PLG82 caused by the initial trapping of oligomers and monomers inside the matrix may lead to a dramatic pH drop after a certain time [25,58], which could induce a robust inflammatory response. Thus, only a matrix which degrades with gradual by-products’ release, such as PLC70, may provide adequate functional properties for FMs’ application. Moreover, this copolymer of L-lactide and ε-caprolactone possesses high elasticity, which is favourable for soft tissue applications [23,24].

## 4. Materials and Methods

PCL (average Mn 80,000, Sigma–Aldrich, UK), P[LAcoCL] with a molar ratio of 70 to 30 (PLC70, RESOMER LC 703 S, Evonic, Essen, Germany), P[LAcoCL] with a molar ratio of 85 to 15 (PLC85, PURASORB PLC 8516, Corbion, Gorinchem, The Netherlands), P[LAcoGA] with a molar ratio of 82 to 18 (PLG82, PURASORB PLG 8218, Corbion, The Netherlands), BaSO_4_ (powder, 0.5 μm, Acros Organics, Geel, Belgium) and HAp (powder, particle size 33 nm, Ca_5_(OH)(PO_4_)_3_, Merck, Darmstadt, Germany) were used in this study.

The compositions of materials are presented in Table 2. The selection of polymers was directed by their similar molecular weights at raw state. The choice of filler content was proceeded by a comparison of the contrasting properties of several material compositions to bone phantom at 100 kV (Figure S1 in Supplementary Materials). Finally, the weight ratio of polymer:BaSO_4_:HAp equal to 8:1:1 was chosen.

Materials were prepared by solvent casting technique as previously [6,13]. Briefly, powders were dispersed by stirring in chloroform (CHCl3, Poch S.A., Gliwice, Poland). Subsequently, the suitable polymer was added and stirred overnight. Solutions were cast to Petri dishes lined with poly(tetrafluoroethylene) (PTFE) foil. The solvent-cast composites were first evaporated under a fume hood at room temperature and then in a vacuum dryer (T = 35 °C, *p* = 100 mbar) for three days. The experimental samples were prepared by thermal extrusion of materials through the G17 nozzle using BioScaffolder (Syseng, Salzgitter-Bad, Germany). The temperatures of the process were presented in Table 2. Fabricated rods 1 mm in diameter were used in further experiments.

### 4.1. Materials Characterisation

#### 4.1.1. Incubation in PBS

A degradation experiment on the extruded rods was performed in phosphate-buffered saline (PBS, Sigma–Aldrich, St. Louis, MO, USA) according to ISO 10993-12. One tablet of PBS was dissolved in 200 mL of demineralized water. The obtained pH was 7.4. The incubation of approx. 50 mg samples in 5 mL PBS was carried out at 37 °C on a shaking platform. PBS solution was changed every two weeks. After 2, 4, 6, 8, 10, 12, 16, 20, and 24 weeks (named W2, W4, etc.), the appropriate samples (three for each time point) were washed in demineralized water, weighted, and dried. The water absorption and mass loss were calculated according to the following equations:

(1) $$\mathrm{Mass}\;\mathrm{loss}=100\%\cdot \frac{\left(m_{0}-m_{d}\right)}{m_{0}}$$

(2) $$\mathrm{Water}\;\mathrm{absorption}\;=100\%\cdot \frac{\left(m_{w}-m_{d}\right)}{m_{d}}$$

where $m_{0}$—a mass of the sample before degradation [mg], $m_{d}$—a mass of the sample after degradation [mg], $m_{w}$—a mass of wet sample [mg]. Masses were measured with an accuracy of 0.01 mg.

#### 4.1.2. MicroCT

Samples of all investigated composites were scanned using SKYSCAN 1174 (Bruker, Kontich, Belgium) before and after 12 and 24 weeks of incubation in PBS at 37 °C. The scanner was set at 40 kV and 250 mA. The scan was performed over 180 degrees with a rotation step of 1°. The obtained planar images were reconstructed with the instrument software, and the image pixel size was 12.67 µm. The 8-bit bitmap (BMP) images represented the attenuation values in a range of 0–7000 HU (at default software calibration). The ImageJ 1.52a software (NIH, Bethesda, MD, USA) was used to define the changes in the attenuation of X-rays during hydrolytic degradation. The average grey value (GV) was measured from a cross-section of rods (*n* = 5).

#### 4.1.3. Mechanical Testing

The elastic modulus (E) of the composite rods was investigated in wet conditions simulating the physiological conditions. The E was determined by a static tensile test on the Dynamic Mechanical Analysis (DMA) instrument Q800 (TA Instruments, New Castle, DE, USA) at 37 °C in PBS. Before the experiment, the fixed ends of samples were embedded in the two-component epoxy adhesive. Then, samples were incubated for 10 min (W0) and two weeks (W2) in PBS at 37 °C with shaking. For the PLG82-1010-BH additional timepoint, one week (W1) was investigated. After that, the tensile test was performed with a rate of 1%/min of the initial length (L0 = 10 mm). The tensile elastic modulus ($E_{t}$) was calculated according to ISO-527 norm using linear regression in the initial quasi-linear range of the stress–strain curve. 

#### 4.1.4. Water Contact Angle Measurements 

The surface wettability was assessed by static water contact angle (WCA) measurements performed on the OCA 20 goniometer (Dataphysics, Filderstadt, Germany). The volume of a sessile droplet was 1 µL. The rods were placed perpendicular to the light–camera axis. The average WCA for each droplet was used in the analysis. There were three spots made for each of the 2 cm-long rods. Fiver rods were tested for each material.

#### 4.1.5. SEM

SEM was performed to observe the surface of fabricated composite rods before and during degradation. Observations were conducted on the Phenom ProX microscope (Phenom-World, Eindhoven, The Netherlands). The acceleration voltage was 10 kV, and an integrated backscattered electron detector (full mode) was used. The detector used in the microscope provides material contrast and topographic imaging in parallel, which was helpful in the analysis of measured samples.

#### 4.1.6. GPC 

The molecular weight (MW) of the polymeric phase of composite samples was assessed by GPC at each step of the composite preparation, after thermal processing as well as before and after each time point of incubation in PBS. The aliquots were prepared by dissolving materials in high-performance liquid chromatography (HPLC) grade chloroform for HPLC (POCH S.A., Gliwice, Poland). Subsequently, centrifugation at 9500× *g* for 10 min and filtration using a PTFE membrane with a pore size of 0.22 µm was performed to remove the inorganic particles. Obtained polymer solutions at a concentration of 2 mg/mL were used in an autosampler of GPC equipment. Aliquots of 100 µL were injected into the system and separated on two linear coupled SEC columns (PLgel 5 mm MIXED-C, UK, 300 × 7.5 mm) at 35 °C and a flow rate of 0.7 mL/min. The molecular weight of the samples was measured using a refractive index detector (Agilent, Darmstadt, Germany). The system was calibrated using nine polystyrene standards (Agilent, Lakeside, UK) with known molecular weights (Mp ranging from 580 g/mol to 990 500 g/mol).

#### 4.1.7. TGA

The filler content was determined using TGA Q5000 (TA Instruments, New Castle, DE, USA). The heating process was performed at 10 °C/min up to 600 °C under an N_2_ atmosphere. The percentage of residual mass after pyrolysis corresponds to the filler content in the composites.

#### 4.1.8. DSC

Differential scanning calorimeter Q2000 (TA Instruments, New Castle, DE, USA) was used to determine the thermal properties of the materials after the composites’ preparation and thermal processing. Moreover, characteristic temperatures and enthalpies were assessed in terms of microstructural changes caused by the degradation of materials.

Measurements were performed at a 10 °C/min rate during the cooling–heating–cooling–heating cycles under the N_2_ atmosphere (50 mL/min flow). The glass transition temperature ($T_{g}$) was determined as an inflexion point, and the melting and cold crystallisation temperatures ($T_{m}$ and $T_{ccr}$) were determined as the maximum of the endo- and exothermic peaks, respectively. The enthalpy of the crystalline phase in the polymeric matrix of the sample before the current heating run was calculated as $\Delta H/w=\left(\sum \Delta H_{m}-\sum \Delta H_{ccr}\right)/w$, where w is the content of polymer (w = (100-Residue at 600 °C)/100) in the sample.

#### 4.1.9. Statistical Analysis

A post hoc one-way ANOVA with a Tukey–Kramer pair-wise comparison test of the results for all subgroups was performed for statistical analysis. A significant difference was noted when the *p*-value was <0.05. 

## 5. Conclusions

The presented study comprehensively compares four composites with PCL, PLC70, PLC85, and PLG82 matrixes for radiopaque FMs’ manufacturing. The obtained data suggest that the most favourable properties were found in the PLC70-based composite, which possessed less stiffness than the PLC85-based composites due to the larger content of soft caprolactone units. Moreover, its contrasting properties were maintained within the required time as no enormous swelling occurred, in contrast to PLG82-composite. The progressing degradation and erosion observed in the PLC70-based composite indicated the forthcoming equable decomposition of the composite, which was needed from the analysed application point of view, and did not occur the in case of the PCL-based composite. The performed experiments enabled in vitro characterization and a comparison of the chosen materials; nevertheless, further examination of the proposed composite material should include in vivo imaging.

## Figures and Tables

**Table 1:** The results of the characterisation of materials in the form of: P—raw polymer, C—solvent cast composite plate, R—thermally extruded rods. TGA: filler content; GPC: *M_n_*—number average molecular weight, *M_w_*—weight average molecular weight, PDI—polydispersity index; DSC (second heating): $T_{g}$—transition temperature, $T_{ccr}$—cold crystallisation temperature, $H_{ccr}$—cold crystallisation enthalpy, $T_{m}$—melting temperature, $H_{m}$—melting enthalpy, ΔH/w—enthalpy of crystalline phase in the polymeric matrix of the sample, w—content of polymer in the sample.

Material	Form	Filler Content ± SD [%]	*M_n_* [kDa]	*M_w_* [kDa]	PDI	$T_{g}$ [°C]	$T_{ccr}$ [°C]/$\Delta H_{ccr}$ [J/g]	$T_{m}$ [°C]/$\Delta H_{m}$ [J/g]	ΔH/w [J/g]
PCL-1010-BH	P		125.7	229.0	1.8	−61.6	-	57.9/59.3	59.3
	C	19.9 ± 0.2	126.8	198.8	1.6	−63.0	-	56.2/51.7	64.5
	R	19.8 ± 0.6	120.1	195.9	1.6	−62.9	-	57.0/53.5	66.6
PLC70-1010-BH	P		99.1	173.4	1.7	30.3	128.0/1.7	159.8/1.8	0.1
	C	19.7 ± 0.4	84.7	161.9	1.8	30.1	106.4/14.8	158.8/15.4	0.7
	R	20.1 ± 1.2	64.2	142.2	2.2	26.9	89.8/17.2	160.0/18.6	1.8
PLC85-1010-BH	P		71.9	228.4	3.2	42.5	-	-	0.0
	C	19.3 ± 0.6	73.7	228.3	3.1	42.1	-	-	0.0
	R	19.9 ± 0.3	58.8	207.0	3.5	42.6	-	-	0.0
PLG82-1010-BH	P		122.8	220.2	1.8	58.7	-	-	0.0
	C	19.8 ± 0.5	125.2	227.7	1.8	57.0	-	-	0.0
	R	19.8 ± 0.4	56.5	151.1	2.7	54.6	-	-	0.0

**Table 2:** Compositions of materials.

Material	Matrix	Composition [%wt]			Temperature of Extrusion [°C]
		Polymer	BaSO_4_	HAp	
PCL-1010-BH	PCL 80 kDa	80	10	10	150
PLC70-1010-BH	RESOMER LC 703 S	80	10	10	190
PLC85-1010-BH	PURASORB PLC 8516	80	10	10	165
PLG82- 1010-BH	PURASORB PLG 8218	80	10	10	200

## Data Availability

Data is contained within the article or supplementary material.

## Supplementary Materials

The following supporting information can be downloaded at: https://www.mdpi.com/article/10.3390/ijms232214363/s1, Section S1: Supplementary tables and figures: Figure S1: The HU value calculated from 8-bit images of reconstructed cross-sections of MicroCT-scanned samples; Figure S2: The DSC thermograms (second heating run): (a) PCL-, (b) PLC70-, (c) PLC85-, and (d) PLG82-based materials in the form of P—raw polymer, C—solvent cast composite plate, R—thermally extruded rods; Figure S3: The SEM image of 3D-printed PCL-1010-BH structure with G32 nozzle (ID 0.1 mm); Figure S4: Thermograms of investigated composite materials within degradation experiment; Figure S5: The characteristic temperatures and enthalpies appointed from second heating run of DSC; Section S2: Detailed DSC analysis; Section S3: FTIR analysis of filler–polymer interactions; Figure S6: The FTIR spectra of: the upper part of the image—solvent cast composite plates (PCL-1010-BH-C (1-yellow), PLC70-1010-BH-C (2-blue) and PLG82-1010-BH-C (3-green)); Figure S7: The FTIR spectra of HAp and BaSO_4_ powders after stirring in CHCl3, PLC70 casted film, and HAp and BaSO_4_ obtained after dissolution of PLC70-based composites containing only one filler; References [64,65,66,67] are cited in the supplementary materials.
