# Supplementary material for: Long-Term In Vitro Assessment of Biodegradable Radiopaque Composites for Fiducial Marker Fabrication
Source: Int J Mol Sci. 2022 Nov 18;23(22):14363. doi: 10.3390/ijms232214363 (PMC9697335; doi:10.3390/ijms232214363)
Supplement: Supplementary file 1 [file ijms-23-14363-s001.zip › ijms-1955551-supplementary.pdf]

International Journal of
Molecular Sciences

MDPI

Supplementary Materials

Long-Term In Vitro Assessment of Biodegradable Radiopaque Composites for Fiducial Marker Fabrication

1. Supplementary Tables and Figures

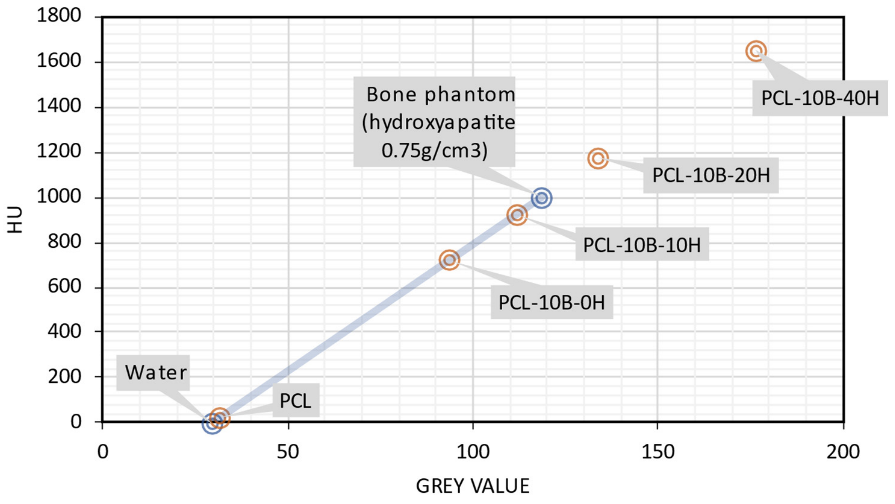

Figure S1. The theoretical HU value calculated from 8-bit images of reconstructed cross-sections of micro CT scanned samples. The scan was performed at 100 kV. The common HU values for Water and Bone were used (0 and 1000, respectively). The tested samples - PCL-based 1-mm rods, were: pure PCL (PCL) and composites with the weight ratio of polymer:BaSO4:HAp equal to: 9:1:0 (PCL-10B-0H), 8:1:1 (PCL-10B-10H), 7:1:2 (PCL-10B-20H), 5:1:4 (PCL-10B-40H). Prepared based on GV data published in [1].

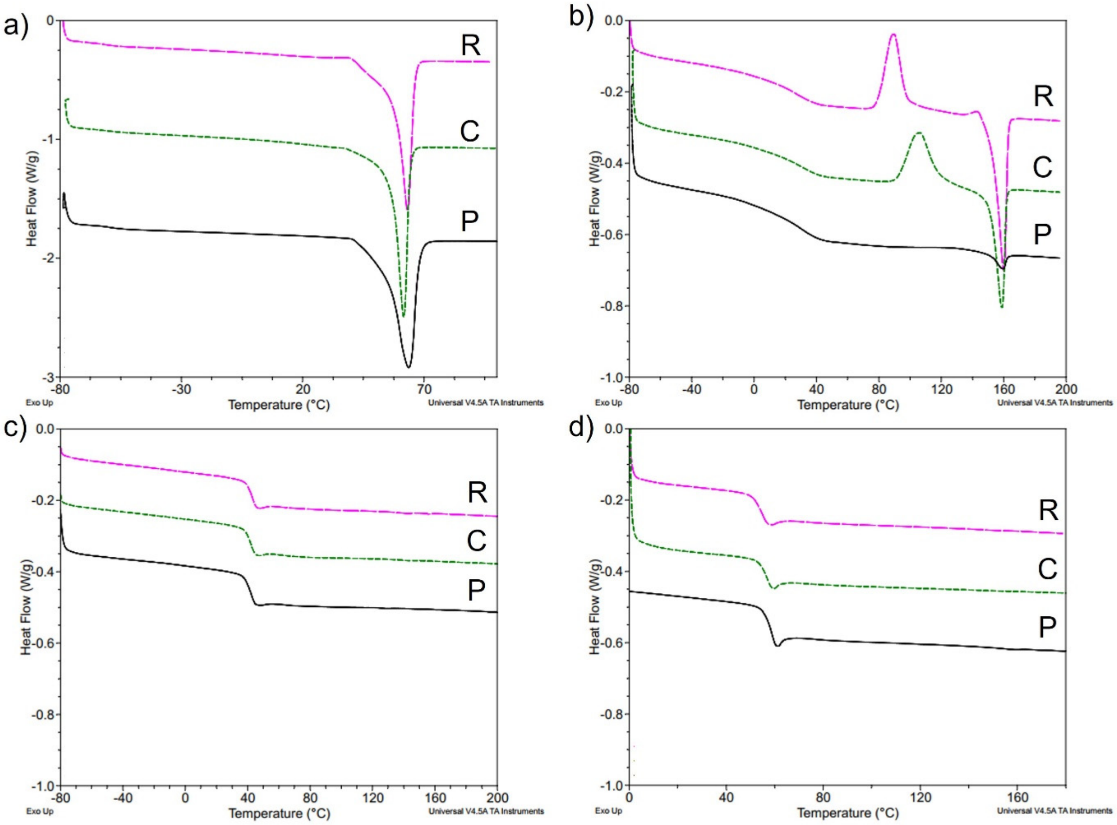

Figure S2. The DSC thermograms (2nd heating run): a) PCL-, b) PLC70-, c) PLC85-, and d) PLG82-based materials in the form of P – raw polymer, C – solvent cast composite plate, R – thermally extruded rods.

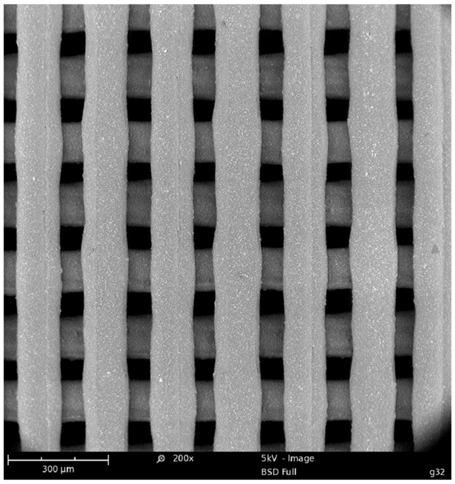

Figure S3. The SEM image of 3D-printed PCL-1010-BH structure with G32 nozzle (ID 0.1 mm).

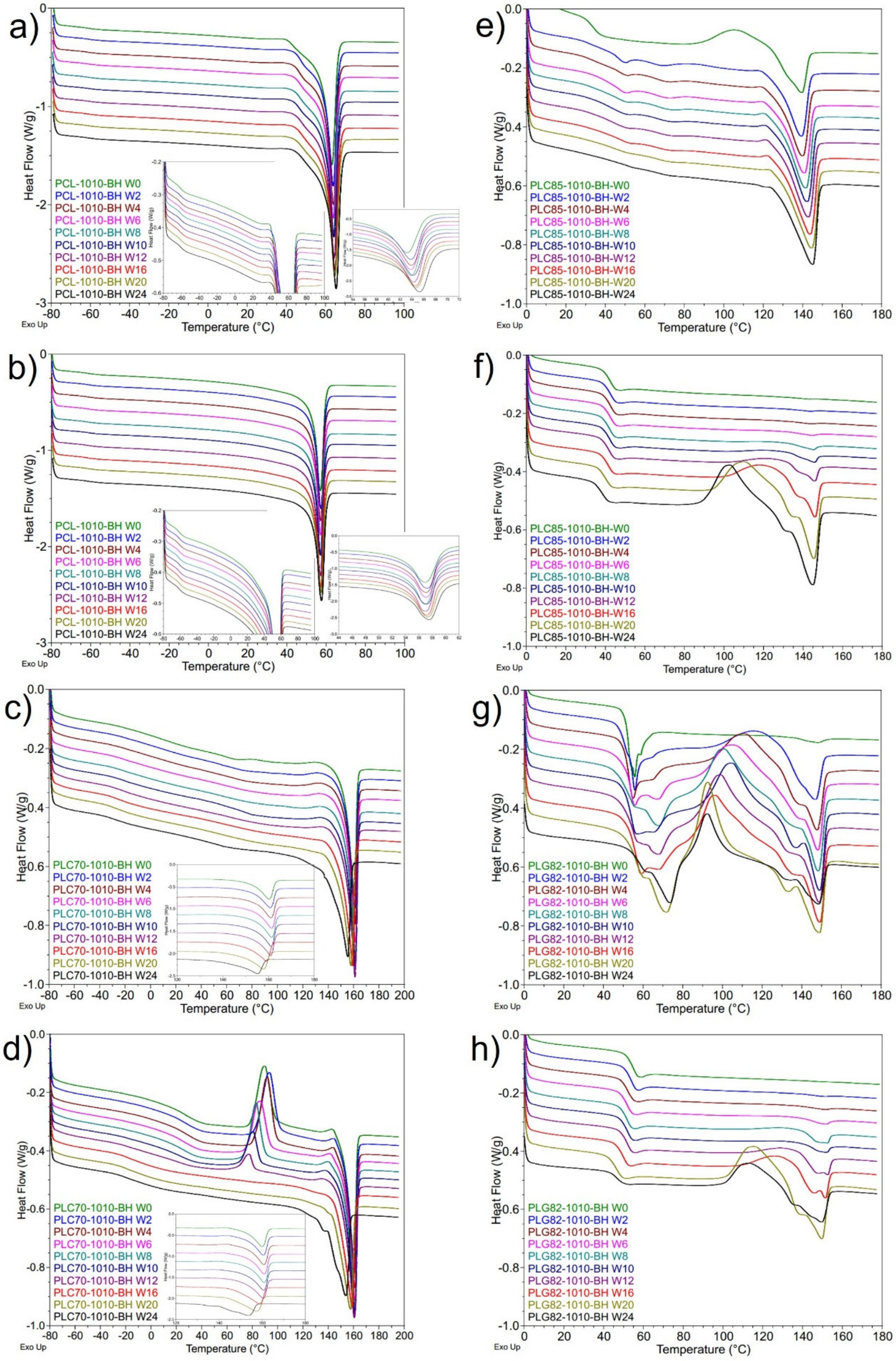

Figure S4. Thermograms of investigated composite materials within degradation experiment (W0, W2, etc. means week 0, week 2, etc.): from 1st (a, c, e, g) and 2nd (b, d, f, h) heating runs; PCL-1010-BH (a, b), PLC70-1010-BH (c, d), PLC85-1010-BH (e, f), PLG82-1010-BH (g, h).

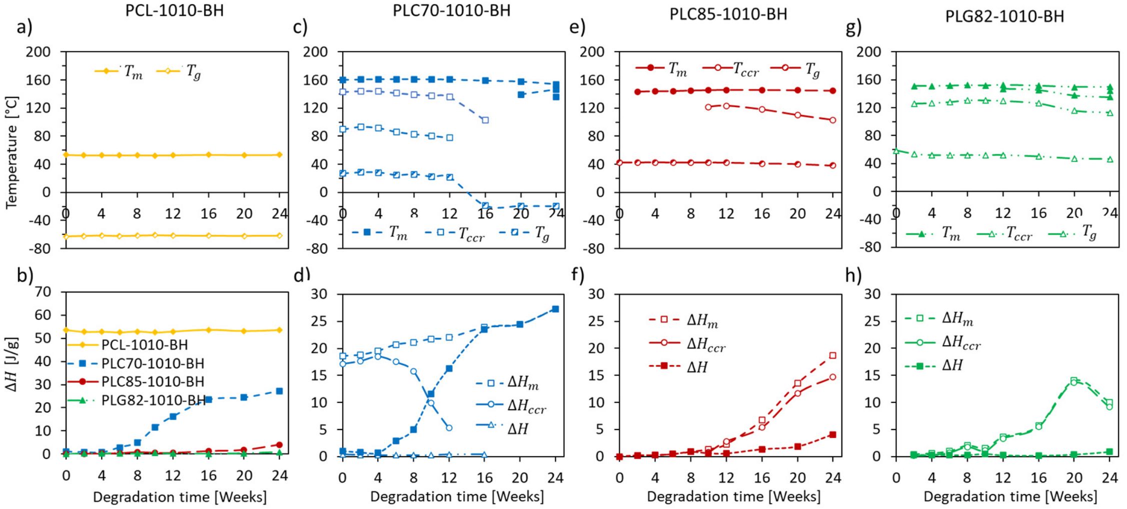

Figure S5. The characteristic temperatures (a, c, e, g) and enthalpies (b, d, f, h) determined from 2nd heating run of DSC for: a) and b) PCL-1010-BH, c) and d) PLC70-1010-BH, e) and f) PLC85-1010-BH, g) and h) PLG82-1010-BH.

2. Detailed DSC Analysis

PLC70-1010-BH

PLC70-1010-BH composite matrix was in a rubbery state at the human body temperature, which was evidenced in the 1st heating cycle of DSC (Figure 6c in the Manuscript and Figure S4c). Two T_g were recorded at W0 - the first at 60°C, originated from lactide segments, changed only slightly during the experiment; the second, linked with caprolactone-containing segments, was found at 7.5°C, then dropped to - 20°C at W4 and remained almost constant till W12. Then, it steadily decreased to - 30°C at W24. These declines reflected the increase of polymeric chains mobility caused by the scission thereof. [2] The matrix of rods of PCL70-based composite crystallised during rods fabrication; the melting peak was found at approx. 160°C at the initial state. Its initial degree of crystallinity was approx. 35%.

Within the degradation experiment, T_m slightly shifted to lower temperatures because of the increased mobility of shorter chains. Parallelly, the growing trend of $H_m$value was also recorded (Figure 6d in the Manuscript). In this DSC run, only a little two peaks of cold crystallisation with maxima at 81.5°C (H_{ccr1} approx. 0.5 J/g) and at 130.5°C (H_{ccr2} approx. 1 J/g) were designated at W0 and disappeared at W2 and W16, respectively.

In the 2nd cycle of heating of the PLC70-1010-BH composite, two cold crystallization peaks were recorded (Figure S4d, Figure S5c,d), but at higher temperatures than in the 1st heating run. Their enthalpies gradually decreased; after W12, the cold crystallization was undetectable. The T_m was found at a similar value as in the 1st heating run. However, a more complex shape of the melting peak was recorded from W20. The respective changes in enthalpies were presented in Figure S5d.

PLC85-1010-BH

The results of the 1st cycle of heating in DSC of PLC85-based composite rods were presented in Figure 6e,f in the Manuscript and Figure S4e. A single glass transition (T_{g1} at 34°C), clear cold crystallisation (T_{ccr} *at* 106°C; H_{ccr} approx. 8 J/g) and melting (T_m at 139.5°C; H_m approx. 9.5 J/g) were observed in the initial state. Thus, the composite matrix was partially in a rubbery state but possessed an amorphous microstructure. However, from W2, the second glass transition (T_{g2} at 45.5°C) and two almost negligible cold crystallisation peaks were found at lower temperatures - T_{ccr1} at 56.5°C and T_{ccr2} at 84°C. Within the experiment, both T_g were almost maintaining its initial value. At W24, T_{g1}

decreased to 33°C and T_{g2} increased to 51°C because of -co- bonds scissions [2,3] and reflecting the changes in the soft (caprolactone) and hard (L-lactide) segments mobility and reorganisation.[4] Within degradation T_m was gradually increasing to 145°C and was recordable during the whole experiment. The lowest H_m was observed at initial state, then it increased evidently as presented in Figure 6f in the Manuscript.

In the 2nd cycle of heating of PLC85-1010-BH composite (Figure S4f, Figure S5e,f), no first-order phase transitions were detected in the initial state. Single T_g (approx. 42.5°C) present in that state was almost constant till W10. Then it slightly decreased. The almost negligible peak of melting (H_m less than 0.5 J/g) was observed till W6, then it was more pronounced. However, from W8 the value of H_m was affected by the crystalline phase formed during cold crystallisation. This also proved the increased mobility of polymeric chains due to material degradation.[4]

PLG82-1010-HB

The results of 1st cycle of heating in DSC of PLG82-1010-BH rods were presented in Figure 6,h in the Manuscript and Figure S4g. The PLG82-based composite showed an almost amorphous structure after rods fabrication. Moreover, the PLG82 matrix was in a glassy state within the degradation experiment. T_g was observed at 52.5°C, with a strong relaxation peak containing two little overlapping endothermic peaks, plausibly originating from melting of the arranged low-MW fraction like monomers and oligomers. [5] The T_g progressively increased to 58.5°C within the experiment, and the overlapping endothermic peaks became more prominent, and the maxima thereof shifted to higher temperatures. Two almost negligible cold crystallisation peaks were also observed in the initial state. However, only the peak at higher temperatures rose and gradually shifted to lower temperatures with time. From W2, its enthalpy changed in a range of 10 - 18 J/g, as shown in Figure 6h in the Manuscript. Parallelly, the present at W0 endothermic peak (H_m 1 J/g) with a maximum at 148°C increased remarkably to 23.5 J/g at W20. However, from W2 this endothermic peak started to demonstrate bimodal characteristics. Within the experiment, the main maximum of the peak remained almost constant, but the new peak recorded at 140°C gradually shifted to 131.5°C at W24. Nevertheless, the total melting peak area largely compensates for the cold crystallisation peak (Figure 6h in the Manuscript). The designated value of ΔH demonstrate the little increase of crystallinity of the PLG82 matrix in the experimental conditions after W12. However, the progressing degradation led to a decrease in its value at the last time interval.

The analysis of the 2nd heating cycle of PLG82-1010-HB (Figure S4h, and Figure S5g,h) revealed difficulty in matrix crystallisation during the cooling run. Only T_g at 58.5°C in the initial state as recorded, which gradually decreased to 46°C at W24 due to MW reduction.[2] Moreover, no overlapping peaks with glass transition were detected in this run. At thermograms of W2 and W4, the little melting peaks were detected with maxima (T_{m1}) at 151°C. Then, the melting peak maximum slightly shifted to higher temperatures and at W12 approached 152.5°C, then steadily decreased to 149.5°C at W24. From W8, the bimodal character of the melting peak appeared with the second maximum (T_{m2}) at 147°C. Even though the main peak was almost stable, the overlapping peak shifted gradually to 135.5°C at the end of the experiment. At the last time point, the additional peak maximum was detected at 144.5°C. From W6, the H_m was gradually increasing to 5.5 J/g at W16, then a sudden rise to 14 J/g occurred at W20, after which H_m decreased to approx. 10 J/g at W24. From W2, the cold crystallisation peak was observed just before the melting peak. With time, its maximum position (T_{ccr}) was increasing from 126°C to 130.5°C at W8, then it was gradually decreasing to 113°C till the end of the experiment. However, the endothermal peak area largely compensated for the exothermal peak area at each time point.

3. FTIR Analysis of Fillers-Polymers Interactions

The interaction of fillers with polymeric chains was assessed utilizing FTIR. The samples of fillers embedded in the matrixes were prepared by the dissolution of 200 mg of composite samples in 4.5 ml of chloroform. The washing procedure was applied after centrifugation in a 5ml Eppendorf tube (1750 x g, 5 min) and the removal of the supernatant. Briefly, fresh chloroform was added, and the tube was shaken for 2 minutes; subsequently, the supernatant was removed. The washing was repeated three times. Obtained powders were initially dried under a fume hood overnight and for three days in a vacuum drier (25ºC, 50 mbar). The infrared spectra were recorded using Nicolet 8700 FTIR spectrometer. Measurements in ATR mode with diamond crystal were made with a resolution of 4 cm^{-1} and 32 scans. Data were collected and analysed in the dedicated Omnic software.

The FTIR analysis indicated the interaction between polymeric chains and fillers in prepared materials (Figure S 6 and Figure S7). The carbonyl band in the ester structure of tested matrixes has an intense stretching signal at 1720 – 1760 cm^{-1}, depending on the material [6,7]. The appropriate peaks were also observed for powders which were obtained after composite matrixes dissolution, proving the presence of polymeric chains on the surface of fillers because neither $BaSO_4$ nor HAp has the peak in this region. Moreover, new, relatively strong peaks were observed, with maxima at 1590-1610 cm^{-1}. These peaks originated from the redshift of the carbonyl band which entered into the interaction with Ca^{2+} or Ba^{2+} ions present at the surface of the fillers [8–10]. Thus, as the attached polymeric chains contained many carbonyl groups, the presence of characteristic carbonyl band in the spectra of powders after matrixes dissolution was justified.

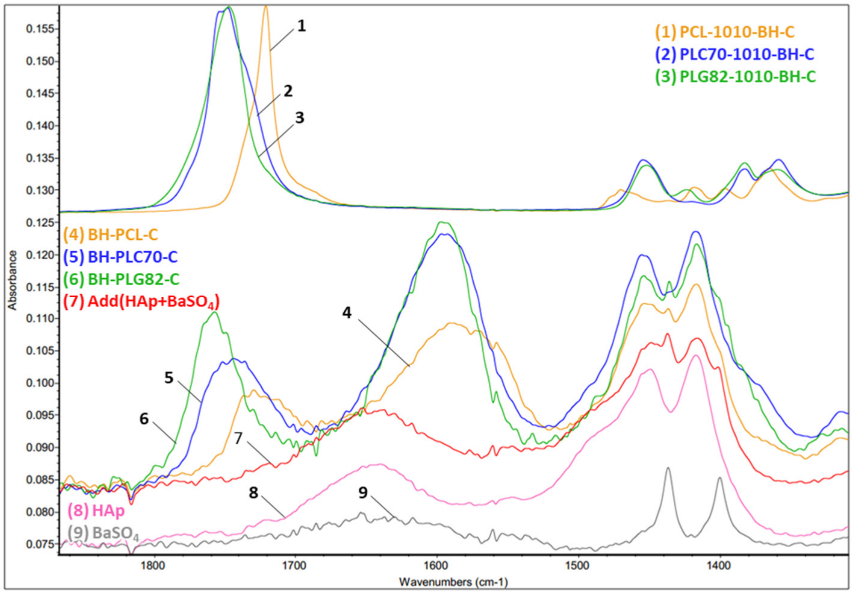

Figure S6. The FTIR spectra of: the upper part of the image - solvent cast composite plates (PCL-1010-BH-C (1-yellow), PLC70-1010-BH-C (2-blue) and PLG82-1010-BH-C (3-green)); the bottom part – fillers after matrix dissolution of solvent cast composite plates (BH-PCL-C (4-yellow), BH-PLC70-C (5-blue) and BH-PLG82-C (6-green)), HAp (8-pink), $BaSO_4$ (9-grey) and synthetically added spectra of HAp and $BaSO_4$ (7-red).

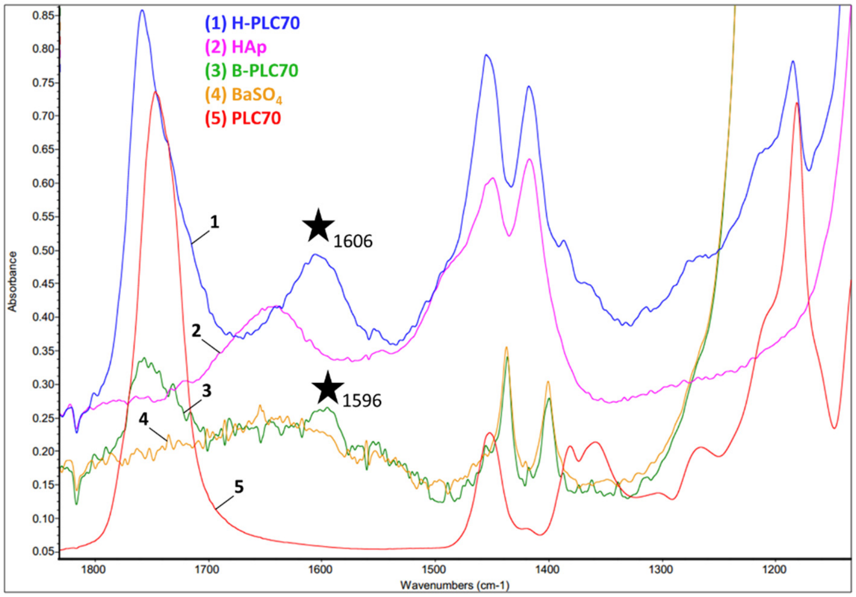

Figure S7. The FTIR spectra of HAp and BaSO4 powders after stirring in $CHCl_3$, PLC70 casted film, and HAp and BaSO4 obtained after dissolution of PLC70-based composites containing only one filler.

References for Supplementary Data

1. Górecka, Ż.; Choińska, E.; Szlązak, K.; Święszkowski, W. Increase of Radiopacity of PCL Scaffolds for Their in Vivo Monitoring Using x – Rays Imaging. *Eur Cell Mater* **2016**, *31*, P207.
2. Zhai, Z.; Morthomas, J.; Fusco, C.; Perez, M.; Lame, O. Crystallization and Molecular Topology of Linear Semicrystalline Polymers: Simulation of Uni- and Bimodal Molecular Weight Distribution Systems. *Macromolecules* **2019**, *52*, 4196–4208, doi:10.1021/ACS.MACROMOL.9B00071.
3. Sivalingam, G.; Madras, G. Thermal Degradation of Binary Physical Mixtures and Copolymers of Poly(ε-Caprolactone), Poly(d, l-Lactide), Poly(Glycolide). *Polym Degrad Stab* **2004**, *84*, 393–398, doi:10.1016/J.POLYMDEGRADSTAB.2003.12.008.
4. Huang, M.H.; Li, S.; Hutmacher, D.W.; Coudane, J.; Vert, M. Degradation Characteristics of Poly(ϵ-Caprolactone)-Based Copolymers and Blends. *J Appl Polym Sci* **2006**, *102*, 1681–1687, doi:10.1002/APP.24196.
5. Rom, M.; Fabia, J.; Slusarczyk, C.; Janicki, J.; Kasperczyk, J.; Dobrzynski, P. Structural Transformation of Terpolymer Poly(L-Lactide-Glycolide- Trimethylene Carbonate) with Shape Memory Effect during the Degradation Process. *Polimery/Polymers* **2014**, *59*, 562–568, doi:10.14314/POLIMERY.2014.562.
6. Pawlik, J.; Łukowicz, K.; Cholewa-Kowalska, K.; Osyczka, A.M. New Insights into the PLGA and PCL Blending: Physico-Mechanical Properties and Cell Response. *Mater Res Express* **2019**, *6*, 085344, doi:10.1088/2053-1591/AB2823.
7. Garkhal, K.; Verma, S.; Jonnalagadda, S.; Kumar, N. Fast Degradable Poly(L-Lactide-Co-ε-Caprolactone) Microspheres for Tissue Engineering: Synthesis, Characterization, and Degradation Behavior. *J Polym Sci A Polym Chem* **2007**, *45*, 2755–2764, doi:10.1002/POLA.22031.
8. Li, Y.; Weng, W. Surface Modification of Hydroxyapatite by Stearic Acid: Characterization and in Vitro Behaviors. *Journal of Materials Science: Materials in Medicine 2007 19:1* **2007**, *19*, 19–25, doi:10.1007/S10856-007-3123-5.
9. Park, J.W.; Hwang, J.U.; Back, J.H.; Jang, S.W.; Kim, H.J.; Kim, P.S.; Shin, S.; Kim, T. High Strength PLGA/Hydroxyapatite Composites with Tunable Surface Structure Using PLGA Direct Grafting Method for Orthopedic Implants. *Compos B Eng* **2019**, *178*, 107449, doi:10.1016/J.COMPOSITESB.2019.107449.
10. Longlade, J.; Delaite, C.; Schuller, A.-S.; Longlade, J.; Delaite, C.; Schuller, A.-S. Surface Modification of Barium Sulfate Particles. *Materials Sciences and Applications* **2021**, *12*, 1–14, doi:10.4236/MSA.2021.121001.
